# Discovery of Non-peptide Small Molecule Allosteric Modulators of the Src-family Kinase, Hck

**DOI:** 10.3389/fchem.2019.00822

**Published:** 2019-11-28

**Authors:** Heather R. Dorman, David Close, Bentley M. Wingert, Carlos J. Camacho, Paul A. Johnston, Thomas E. Smithgall

**Affiliations:** ^1^Department of Microbiology and Molecular Genetics, University of Pittsburgh School of Medicine, Pittsburgh, PA, United States; ^2^Department of Pharmaceutical Sciences, University of Pittsburgh School of Pharmacy, Pittsburgh, PA, United States; ^3^Department of Computational and Systems Biology, University of Pittsburgh School of Medicine, Pittsburgh, PA, United States

**Keywords:** Src-family kinase, allosteric action, SH2 and SH3 domains, Hck kinase, high throughput screening, fluorescence polarization assay

## Abstract

The eight mammalian Src-family tyrosine kinases are dynamic, multi-domain structures, which adopt distinct “open” and “closed” conformations. In the closed conformation, the regulatory SH3 and SH2 domains pack against the back of the kinase domain, providing allosteric control of kinase activity. Small molecule ligands that engage the regulatory SH3-SH2 region have the potential to modulate Src-family kinase activity for therapeutic advantage. Here we describe an HTS-compatible fluorescence polarization assay to identify small molecules that interact with the unique-SH3-SH2-linker (U32L) region of Hck, a Src-family member expressed exclusively in cells of myeloid lineage. Hck has significant potential as a drug target in acute myeloid leukemia, an aggressive form of cancer with substantial unmet clinical need. The assay combines recombinant Hck U32L protein with a fluorescent probe peptide that binds to the SH3 domain in U32L, resulting in an increased FP signal. Library compounds that interact with the U32L protein and interfere with probe binding reduce the FP signal, scoring as hits. Automated 384-well high-throughput screening of 60,000 compounds yielded Z'-factor coefficients > 0.7 across nearly 200 assay plates, and identified a series of hit compounds with a shared pyrimidine diamine substructure. Surface plasmon resonance assays confirmed direct binding of hit compounds to the Hck U32L target protein as well as near-full-length Hck. Binding was not observed with the individual SH3 and SH2 domains, demonstrating that these compounds recognize a specific three-dimensional conformation of the regulatory regions. This conclusion is supported by computational docking studies, which predict ligand contacts with a pocket formed by the juxtaposition of the SH3 domain, the SH3-SH2 domain connector, and the SH2-kinase linker. Each of the four validated hits stimulated recombinant, near-full-length Hck activity *in vitro*, providing evidence for allosteric effects on the kinase domain. These results provide a path to discovery and development of chemical scaffolds to target the regulatory regions of Hck and other Src family kinases as a new approach to pharmacological kinase control.

## Introduction

Acute myeloid leukemia (AML) is a hematologic cancer correlated with advancing age and often poor prognosis (Dohner et al., 2015; Khwaja et al., 2016). While many genetic changes contribute to AML, enhanced signaling by both receptor and non-receptor tyrosine kinases is a common theme that presents opportunities for drug therapy. One notable example involves mutations in Flt3, a receptor tyrosine kinase normally involved in the regulation of myeloid progenitor cells in the bone marrow (Kayser and Levis, 2014; Coombs et al., 2016). Two classes of mutations are associated with ligand-independent Flt3 activation in AML. These include internal tandem duplications (ITDs) in the regulatory juxta-membrane domain of the kinase, and activating point mutations within the kinase domain (Gu et al., 2011; Konig and Levis, 2015). Broad-spectrum ATP-site kinase inhibitors with activity toward Flt3 have been tested in the clinic for AML. Midostaurin and sorafenib exhibited limited efficacy as monotherapy due to a lack of sustained Flt3 inhibition and toxicity associated with off-target kinase inhibition (Wander et al., 2014). More selective inhibitors, including quizartinib, crenolanib, and gilteritinib (Perl et al., 2017), show enhanced specificity for the Flt3 kinase domain and improved efficacy as single agents (Zarrinkar et al., 2009; Zimmerman et al., 2013). However, inhibitor resistance, often involving acquired mutations in the Flt3 kinase domain, may limit their effectiveness (Pratz and Luger, 2014; Sudhindra and Smith, 2014; Konig and Levis, 2015).

Non-receptor tyrosine kinases expressed in myeloid hematopoietic cells have also been implicated in AML pathogenesis, and have attracted attention as alternative targets for kinase inhibitor discovery. Members of the Src kinase family expressed in myeloid cells, including Hck, Fgr, and Lyn, are particularly attractive candidates for drug development. Knockdown of each of these Src family members individually suppressed the growth of patient-derived AML cells *in vitro* (Dos Santos et al., 2013). Hck transcripts are enhanced in gene expression profiles of myeloid leukemia stem cells relative to normal bone marrow progenitor cells (Saito et al., 2010). Furthermore, an ATP-site kinase inhibitor with activity against Hck known as A-419259 (Wilson et al., 2002) suppressed the growth of patient AML cells in engrafted immunocompromised mice (Saito et al., 2013), although subsequent studies have shown that this compound is also active against Fgr and Lyn as well as Flt3. Lyn is over-expressed and active in most clinical AML isolates and has been linked to the activation of Stat5 by Flt3-ITD (Robinson et al., 2005; Okamoto et al., 2007; Dos Santos et al., 2008). Fgr is also strongly expressed in a subset of AML patient samples regardless of Flt3 mutational status (Shen et al., 2018; Weir et al., 2018). A recent study reported discovery of an ATP-site inhibitor for Fgr with potent anti-AML activity both *in vitro* and in a xenograft model of AML *in vivo* (Weir et al., 2018). Together, these studies support the idea that targeted inhibition of myeloid Src-family members may be a viable therapeutic strategy in AML patients where these kinases are over-expressed and active.

**Figure 1:**
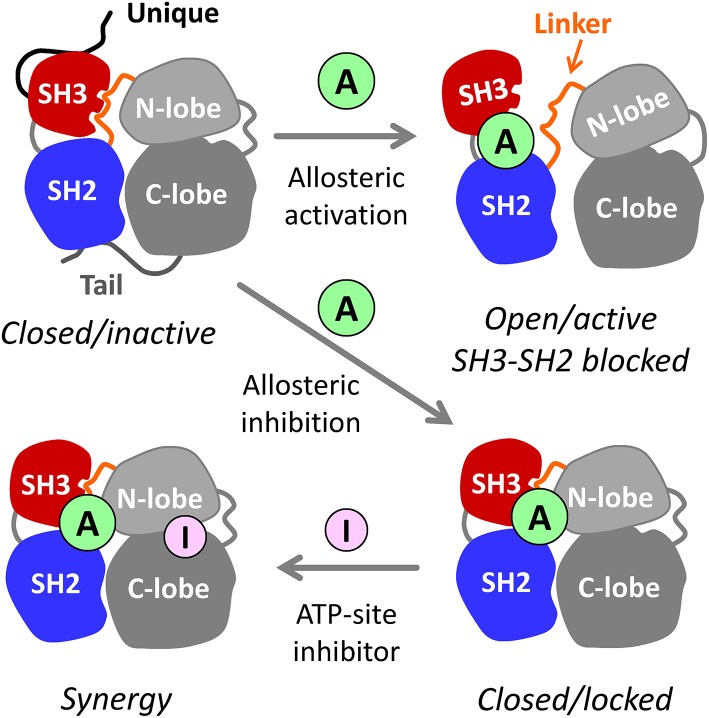
Allosteric modulators of Src-family kinases. Hck, Fgr, and other Src-family kinases are regulated by intramolecular interactions of their SH3, SH2, and bi-lobed kinase domains (N- and C-lobes). The SH3 and SH2 domains pack against the back of the kinase domain to stabilize the inactive, assembled kinase conformation (upper left). Small molecule allosteric ligands (A) that bind to the SH3-SH2-linker region have the potential to disrupt its regulatory influence on the kinase domain, resulting in kinase activation (top right). Because SH3 and SH2 are also involved in *trans*-interactions with other proteins, small molecules that bind to this region may interfere with signaling by altering substrate recruitment. Allosteric ligands also have the potential to stabilize the closed, inactive conformation (lower right). Such compounds may inhibit the kinase directly and/or enhance the potency of existing ATP-site inhibitors (I) that prefer a particular conformation of the active site (lower left—see text for details). For simplicity, the N-terminal unique domain and regulatory C-terminal tail are shown only for the closed/inactive state.

In addition to their kinase domains, Src-family members also share modular SH3 and SH2 domains that function in protein-protein interactions essential for kinase regulation and signaling (Engen et al., 2008). These non-catalytic domains represent an unexplored opportunity for selective inhibitor discovery. In the inactive state, Hck and other Src-family kinases adopt a compact, assembled conformation with the SH3 domain bound to the SH2-kinase linker and the SH2 domain bound to the tyrosine-phosphorylated tail (Figure 1). When the SH3 and SH2 domains are displaced from their negative regulatory positions on the back of the kinase domain, the overall structure becomes more dynamic, assuming multiple three-dimensional conformations (Yang et al., 2010). Release from SH3-SH2 regulatory constraint also allows the kinase domain to adopt the active conformation. As illustrated in Figure 1, small molecules that interact with this region of the kinase may have several impacts on overall kinase structure and function. One possibility is that the ligand may destabilize the closed, inactive conformation, resulting in kinase activation; previous studies have demonstrated this outcome using short peptide ligands that bind to the SH3 or SH2 domains or to both simultaneously (Moroco et al., 2014, 2015). Alternatively, the ligand may stabilize the regulatory interactions observed in the crystal structures of near-full-length Hck and Src, thus representing allosteric kinase inhibitors. In either case, binding of the allosteric ligand may push the kinase into a single conformation, thereby potentiating the activity of ATP-site inhibitors that prefer a given conformation of the active site (Liu and Gray, 2006). Regardless of the effect on kinase activity, tight binding of small molecule ligands to the SH3-SH2 regulatory region may interfere with protein-protein interactions essential for signal transduction.

In this study, we developed a high-throughput screening (HTS) assay based on fluorescence polarization (FP) to identify small molecule, non-peptide ligands that bind directly to the non-catalytic unique-SH3-SH2-linker region of the AML-associated Src-family kinase, Hck. Screening of two diverse chemical libraries totaling 60,000 discrete compounds identified a hit series with a shared pyrimidine diamine substructure. Surface plasmon resonance (SPR) assays confirmed direct interaction of these hit compounds with the multi-domain Hck target protein used in the screen, as well as near-full-length Hck. Interestingly, no interaction was observed with the individual SH3 and SH2 domains. This observation suggests that the compounds recognize structural features only present in the assembled conformation of the kinase, a conclusion supported by computational docking studies with the crystal structure of near-full-length Hck. Incubation of near-full-length, downregulated Hck with these compounds led to kinase activation *in vitro*, providing evidence for allosteric effects on kinase activity. Our results provide a path to discovery of allosteric modulators of Hck and other Src-family members as a new approach to targeted therapy for AML or other neoplastic diseases that over-express these kinases.

## Materials and Methods

### Expression and Purification of Recombinant Kinase Proteins

Recombinant Hck and Fgr SH3, SH2, SH3-SH2, and U-SH3-SH2-linker (U32L) proteins were expressed in *E. coli* and purified as described in detail elsewhere (Lionberger et al., 2000; Lerner and Smithgall, 2002; Shen et al., 2018). Near-full-length Hck-YEEI (Moroco et al., 2014) and Fgr-YEEI (Shen et al., 2018) proteins were expressed in their respective downregulated conformations in Sf9 insect cells using previously published methods. “YEEI” refers to the sequence of the C-terminal tail, which is modified to encode Tyr527-Glu-Glu-Ile; this sequence binds to the SH2 domain with higher affinity than the wild type tail and promotes purification of the kinase in the assembled, inactive conformation (Schindler et al., 1999; Porter et al., 2000).

### Fluorescence Polarization (FP) Assay for High-Throughput Screening (HTS)

The Src-family kinase SH3 domain probe peptide, VSL12, was synthesized by the University of Pittsburgh Genomics and Proteomics Core Laboratories. For the FP assay, the peptide was labeled with 6-carboxyfluorescein at its N-terminus. The K_D_ of the FP probe peptide for the Hck-U32L protein was determined to be 2.6 μM by SPR. Molecular weight and purity of all peptides were verified by mass spectrometry. Stock solutions (10 mM) were prepared in a 1:1 mixture of DMSO and FP assay buffer (20 mM Tris-HCl, pH 8.3) for the labeled peptide and FP assay buffer for the unlabeled peptide. Stock solutions were stored at −20°C.

FP assays were performed in low-volume black 384-well plates with a non-binding surface (Corning #3676). Peptides and proteins were added to each well in FP assay buffer for a final assay volume of 20 μL and mixed by shaking for 5 min at room temperature. The average FP signals derived from ten flashes per well were recorded at an excitation wavelength of 485 nm and emission wavelength of 515 nm in a SpectraMax M5 microplate reader (Molecular Devices) using the Softmax Pro software (version 5.4.1). Raw fluorescence intensity was also read at the same wavelengths for each assay.

Screening libraries were purchased from ChemDiv (10,000 non-peptide peptidomimetic library) and ChemBridge (50,000 compound diversity library). Each library compound was screened at 50 μM and a final DMSO concentration of 1%. Compounds were added to 384-well assay plates first, followed by a pre-mixed complex of the Hck U32L protein (5 μg; 9.4 μM final concentration) and the VSL12 probe peptide (1 μM final concentration). Each plate contained 32 wells of the Hck U32L protein plus probe peptide and DMSO as positive controls, and 32 wells of mutant Hck U32L-W118A protein plus probe peptide and DMSO as negative controls. Results for each compound test well were ranked by z score analysis according to the formula: z = (sample FP—mean FP_samples_)/standard deviation_samples_ (Shun et al., 2011). Potential hit compounds, defined as those that inhibited the FP signal by at least 30% and/or with z scores < −3.0, were then retested in quadruplicate using the FP assay under screening assay conditions.

### Surface Plasmon Resonance (SPR) Assay

SPR analysis was performed on a Reichert 4SPR instrument (Reichert Technologies) using four-channel carboxymethyl dextran hydrogel biosensor chips (Reichert #13206066). Recombinant purified Hck proteins were covalently attached to the chip surface via standard amine coupling chemistry (Grover et al., 2015). Compounds were prepared in 20 mM Tris-HCl, pH 8.3, 150 mM NaCl, and 1% DMSO and flowed past the immobilized proteins and a reference channel at a flow rate of 50 μL/min for 60 s over a range of concentrations. The binding reaction was followed by dissociation for 120 s, followed by surface regeneration using 10 mM HEPES, pH 7.5, containing 150 mM NaCl, 3 mM EDTA, and 0.05% polysorbate 20, at a flow rate of 50 μL/min for 30 s. All sensorgrams were recorded in triplicate, corrected for buffer effects, and fitted with the 1:1 Langmuir binding model using the TraceDrawer software (Reichert).

### Computational Docking Studies

The structures of hit compounds **1** through **4** were docked to the X-ray crystal structure of near-full-length Hck (PDB: 1QCF) using the program Smina (Koes et al., 2013) with default docking parameters. Compounds were first docked to the region defined by the SH3-SH2-linker region, and all four compounds were found to target the same pocket formed at the intersection of these three domains. Compounds were then redocked with higher exhaustiveness at the center of the overall Hck structure plus an outer shell of 8 Å.

### *In vitro* Kinase Assay

The Z'Lyte *in vitro* kinase assay (Life Technologies) was performed in 384-well low volume, non-binding, black polystyrene microplates according to the manufacturer's instructions. The assay reports phosphorylation of a FRET peptide substrate (Tyr2) tagged with coumarin and fluorescein on its N- and C-termini, respectively. Following incubation with the kinase and ATP, reactions were quenched with a site-specific protease that selectively cleaves the unphosphorylated peptide, resulting in loss of the FRET signal. Each kinase was incubated in the presence or absence of the compound for 30 min. Phosphorylation reactions were then initiated by the addition of ATP at the K_m_ for each kinase with the Tyr2 peptide substrate at 1 μM. Following a 1 h incubation, reactions were quenched with the development protease, and coumarin and fluorescein fluorescence were recorded 1 h later on a Molecular Devices SpectraMax M5 plate reader. Data are expressed as a ratio of coumarin fluorescence (445 nm) to the fluorescein FRET signal (520 nM), and normalized to ratios obtained in the absence of ATP (negative control) and with a positive control Tyr2 peptide that is 100% phosphorylated.

## Results and Discussion

### Development and Validation of an FP-Based, HTS Assay for Src-Family Kinase Allosteric Modulator Discovery

**Figure 2:**
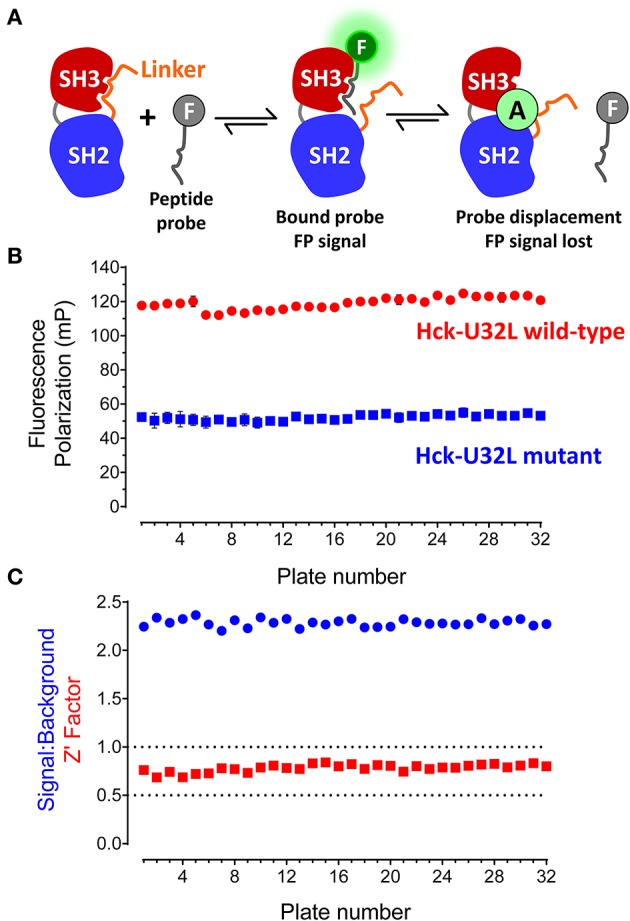
FP-based HTS assay for SH3-SH2 ligands. **(A)** FP assay principle. The FP assay for Hck combines a recombinant N-terminal unique-SH3-SH2-linker (“U32L”) protein with an SH3-binding probe peptide labeled with fluorescein (F). Probe peptide binding to the SH3 domain in the target protein results in an FP signal. Small molecule allosteric modulators (A) may bind to the SH3 domain and block probe peptide binding directly as shown or stabilize SH3:linker interaction, making the SH3 domain inaccessible to the probe peptide (*not shown*). In either case, small molecule binding reduces the FP signal. The N-terminal region is not illustrated for clarity. **(B)** FP assay performance under robotic HTS conditions. The FP assay was automated and used to screen a 10,000-compound library using 32 × 384-well plates. Each plate had 32 wells of the target protein with DMSO only (Hck-U32L wild-type) and 32 wells of a protein with an inactivating mutation in the SH3 domain that cannot bind the probe as the negative control (Hck-U32L mutant). Average FP (mP) values from the control wells are shown ± S.D.; note that some error bars are smaller than the size of the corresponding data point. **(C)** Screening statistics. Signal-to-background ratios and Z'-factor coefficients calculated from control wells on each assay plate are shown.

The target protein for HTS consisted of the entire regulatory region of the p59 form of human Hck, including the N-terminal unique region, followed by the SH3 and SH2 domains, and the SH2-kinase linker (referred to hereafter as the Hck “U32L” protein). A fluorescein-tagged, proline-rich 12-mer peptide known as “VSL12” served as the FP probe (sequence: VSLARRPLPPLP). VSL12 binds to the SH3 domain of Hck and other Src-family kinases with low micromolar affinity (Moroco et al., 2014). The principle of the assay is illustrated in Figure 2A. Binding of the probe peptide to the SH3 domain in the target protein slows its rotation, resulting in an increase in the FP signal. Library compounds that interfere with probe binding or stabilize SH3-linker interaction reduce the FP signal, and score as hits.

The Hck-U32L protein was expressed in *E. coli* and purified via affinity, ion-exchange and gel-filtration chromatography. The purity and identity of the U32L protein were confirmed by SDS-PAGE and mass spectrometry, which showed a single protein species of the anticipated molecular weight (data not shown). As a negative control, we also produced a mutant Hck-U32L protein in which a conserved SH3 domain tryptophan residue in the VSL12 peptide binding site (Trp118; numbering based on the crystal structure of Src (Xu et al., 1999) was substituted with alanine (Feng et al., 1995). The VSL12 peptide probe was tagged at its C-terminus with 6-carboxy-fluorescein. Assay development studies identified the optimal VSL12 FP peptide probe and U32L target protein concentrations, and demonstrated that the resulting FP signal was stable for up to 6 h. Well-to-well variability was then assessed, and yielded a consistent signal to background ratio with low variation, resulting in a Z'-factor coefficient of 0.57 that is compatible with HTS (Zhang et al., 1999). Results of additional assay validation studies are presented in the Supplementary Information file. These results include titration of the FP peptide probe against the Hck-U32L protein target (Figure S1), the titration of the target protein at a fixed concentration of probe (Figure S2), and an assay validation study under HTS conditions of five plates in the presence of DMSO (Figure S3).

Using the automated Hck-U32L FP assay, we performed a pilot screen with a non-peptide, peptidomimetic library of 10,000 discrete compounds. Reactions (20 μL) were performed in 384-well black plates with library compounds at 50 μM, 5 μg of the recombinant Hck-U32L target protein, and 1 μM fluorescent VSL12 probe peptide per well. Each assay plate had 32 positive (DMSO co-solvent only) and 32 negative (Hck-U32L-W118A mutant) control wells to monitor assay performance. The Hck-U32L assay controls yielded very reproducible FP signals, with S/B ratios > 2.0 and Z'-factor coefficients > 0.7 across all 32 assay plates (Figures 2B,C). Based on these favorable pilot screening statistics, we extended the Hck-U32L primary screen to a ChemBridge Diversity Library of 50,000 compounds. This collection is a representative subset of the ChemBridge 410,000-compound core library and is designed to provide the widest coverage of pharmacophore space within 50,000 compounds while maintaining drug-like properties. This screen consisted of 157 compound plates; all plates had S/B ratios ranging from 1.6 to 2.1 with Z'-factor coefficients ranging from 0.7 to 0.9, providing additional evidence for a robust and reproducible assay.

**Figure 3:**
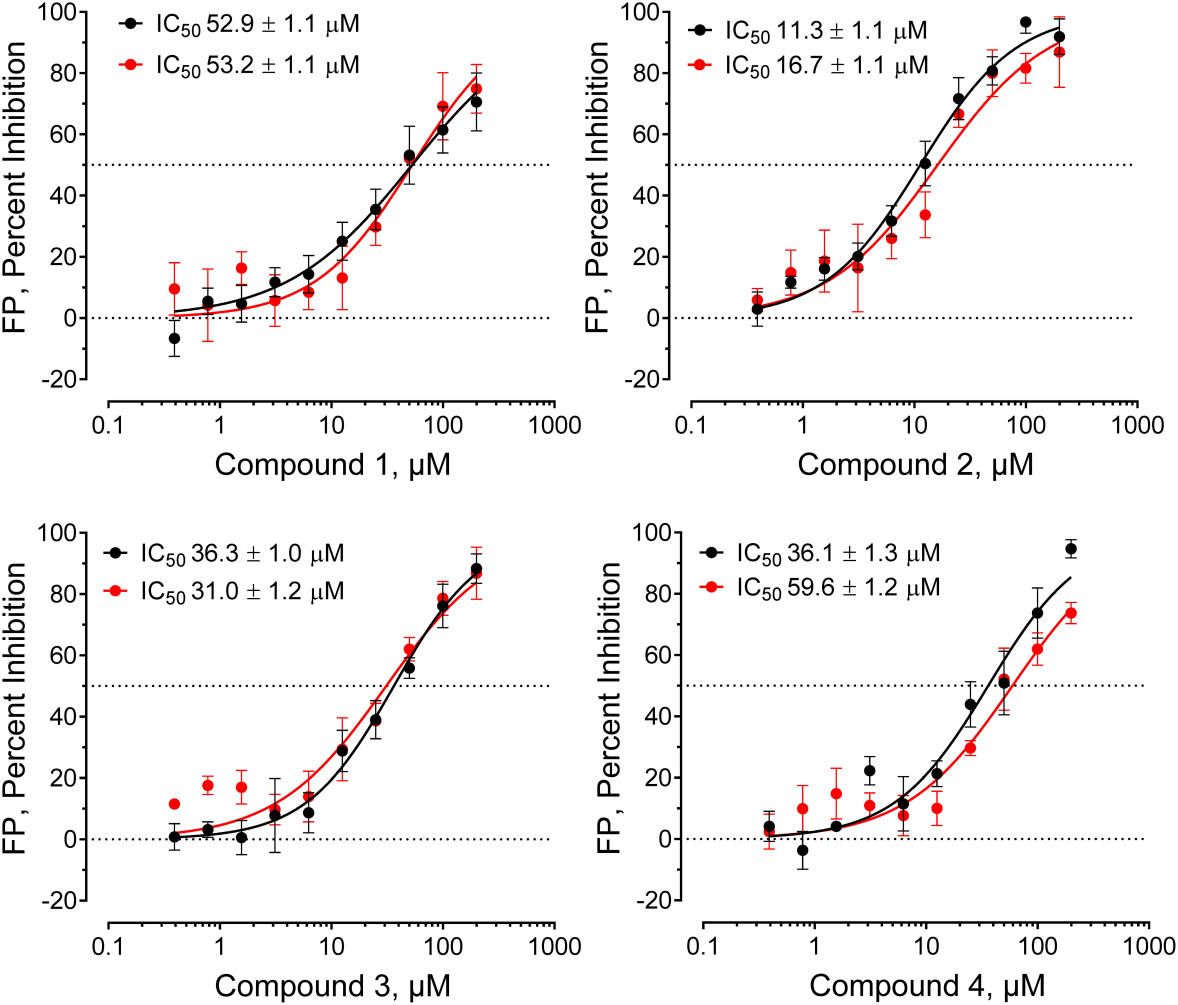
Hck-binding compounds inhibit Hck-U32L target protein interaction with the VSL12 probe peptide in a concentration-dependent manner. Four confirmed hit compounds from the FP screen of the ChemBridge 50,000 compound library were purchased as powders, and re-tested in the screening assay over a wide range of concentrations as shown (*n* = 2; each data point in triplicate). The IC_50_ values from each of the two determinations ± S.E. are shown at the top left in each graph.

**Figure 4:**
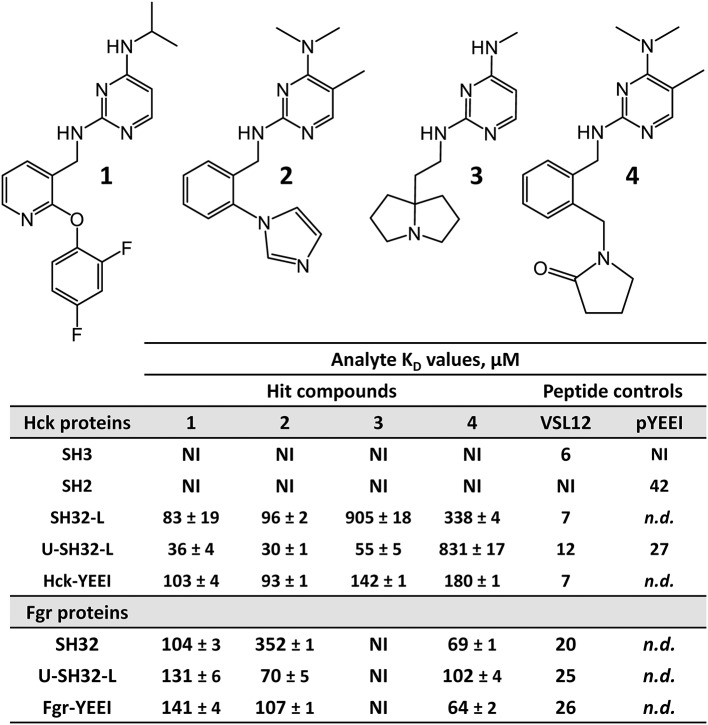
FP-based HTS of the ChemBridge 50K compound diversity set with the Hck-U32L protein identifies four hit compounds with a shared pyrimidine diamine substructure. The structures of the four hit compounds are shown at the top. Each compound was tested for direct interaction with each of the Hck and Fgr proteins listed using SPR as described in the legend to Figure 5. SPR data were best fit by a 1:1 Langmuir model yielding the kinetic K_D_ values shown ± S.E. No binding was observed with the isolated Hck SH3 or SH2 domains. Peptides that selectively interact with the SH3 domain (VSL12) or the SH2 domain (pYEEI) were included as positive controls. NI, no interaction; *n.d*., not determined.

Compounds were considered active if they reduced the FP signal by at least 30% and/or three standard deviations relative to the mean FP value of the 320 compound wells on each plate. This metric is readily calculated as the z-score [z-score = (x–μ)/σ, where x is the value for each compound, μ is the population mean, and σ is the standard deviation for the population]. Forty-nine compounds inhibited probe binding (z-scores ≤ −3.0) for a raw primary HTS active rate of 0.08%. Raw HTS actives were then cherry-picked and re-tested in replicate wells (*n* = 3) using the FP assay in the presence and absence of the Hck-U32L target protein to rule out assay interference. Four compounds were ultimately confirmed as active, and then validated further in concentration-response assays (Figure 3). Each compound showed concentration-dependent inhibition of the VSL12 peptide FP signal in two independent replicate experiments, with IC_50_ values in the 10–50 μM range. Remarkably, all four compounds share a common pyrimidine diamine substructure as shown in Figure 4.

Although the overall hit rate for the screen was quite low (just 49 raw hits out of 60,000 screened compounds or 0.08%), the false positive rate among these raw hits was arguably on the high side (4 confirmed out of 49 raw hits). This outcome relates to the cut-off used to define the raw hits. Preliminary analysis of the HTS data used a cut-off for active hit compounds of 50% inhibition of the FP signal relative to the negative control. Using this more stringent definition, only 14 compounds achieved ≥ 50% inhibition in the primary HTS assay (0.02%). Therefore, we decided to cherry-pick actives that exhibited ≥ 30% inhibition and/or a z-score ≤ −3 as described above. Although we anticipated that lowering the active criterion would likely increase the number of false positives and reduce the confirmation rate, we wanted to cast a wider net to find potential actives. This more relaxed cut-off identified the 49 raw hit compounds selected for confirmation. These compounds were cherry-picked and re-screened alone for auto-fluorescence and in the presence of the FP-peptide probe alone to determine if they augmented or quenched the probe fluorescence intensity. Confirmation assay plates were also pre-read in the molecular polarization mode prior to the addition of the Hck-U32L target protein. Although one of the cherry-picked compounds was auto-fluorescent, it did not affect the FP assay. None of the remaining compounds interfered with either the probe fluorescence intensity or the molecular polarization pre-reads in a concentration-dependent manner. Although the strategy of lowering the active criterion reduced the confirmation rate, it enabled us to identify the four structurally related compounds shown in Figure 4. The straightforward nature of the FP assay allowed for rapid triage and hit confirmation.

### Confirmation of Allosteric Ligand Binding and Protein Target Specificity by SPR

To test whether the hit compounds from the Hck-U32L FP screen interacted directly with the target protein, we performed SPR studies using the Hck-U32L protein from the FP screen along with the individual SH3 and SH2 domains, an SH3-SH2 dual domain protein, and a near-full-length form of Hck consisting of the SH3, SH2 and kinase domains plus the tyrosine-phosphorylated tail (‘Hck-YEEI', where YEEI refers to a sequence modification in the C-terminal tail that facilitates purification in the inactive, assembled conformation (Schindler et al., 1999). The recombinant purified Hck proteins were covalently attached to carboxymethyl dextran biosensor chips, and each hit compound was injected in triplicate over a range of concentrations. Interaction was monitored to equilibrium, followed by a dissociation phase. The resulting sensorgrams were best-fit to determine the kinetic rate constants and corresponding K_D_ values, where K_D_ = k_off_/k_on_. As positive controls, we also monitored the interaction of peptide ligands for the SH3 and SH2 domains. These included the VSL12 peptide for SH3 (same sequence as the peptide probe used for the FP assay), and a tyrosine phosphopeptide known to bind to the Hck SH2 domain (“pYEEI” peptide) (Moroco et al., 2015).

**Figure 5:**
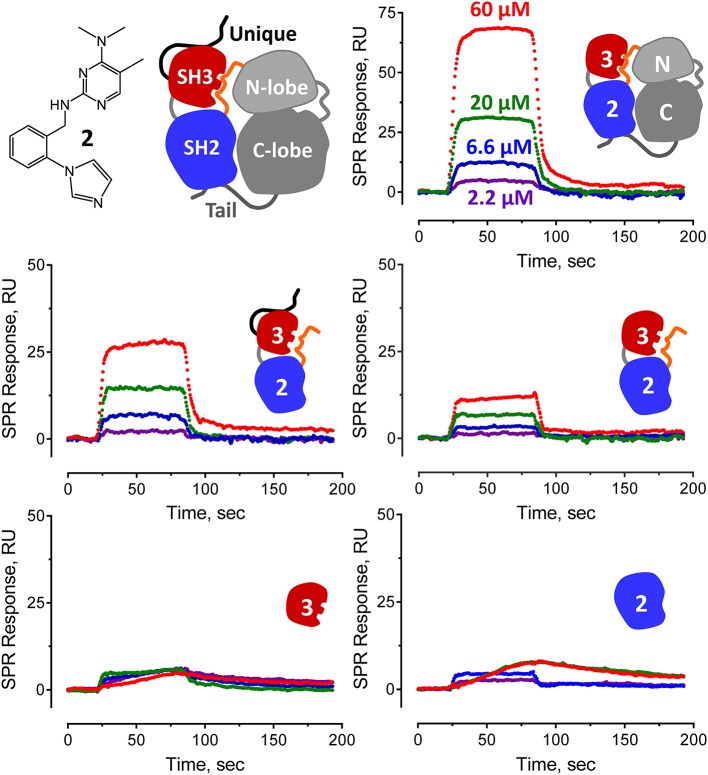
Pyrimidine diamine compound 2 interacts preferentially with multi-domain Hck proteins by SPR. Hit compound **2** from the Hck-U32L FP screen of the ChemBridge 50,000 compound diversity library was tested for direct binding to the five Hck proteins illustrated. A cartoon of inactive full-length Hck is shown for reference (*top left*) along with the structure of compound **2**. Each Hck protein component shown was immobilized on a Reichert carboxymethyl dextran chip at the same density. Compound **2** was injected over the range of concentrations shown, and interaction was monitored to equilibrium followed by a dissociation phase. Sensorgrams were best-fit by a 1:1 binding model to yield kinetic K_D_ values (see Figure 4).

Representative sensorgrams for hit compound **2** are shown in Figure 5, and all SPR data (including control peptide data) are summarized in Figure 4. Compound **2** bound readily to the Hck-U32L protein in a concentration-dependent manner, yielding a K_D_ value of 30 μM. Remarkably, the extent of binding was reduced by about 50% and the K_D_ increased about 3-fold with an SH3-SH2-linker protein lacking the N-terminal unique domain. This observation suggests that the unique region is either directly involved in compound binding, or that it stabilizes a conformation of the SH3-SH2-linker region to favor interaction. Recent NMR studies have shown that the unique domains of other Src-family members interact in an intramolecular fashion with their SH3 domains, consistent with this possibility (Arbesú et al., 2017). No compound binding was observed with the isolated SH3 or SH2 domains alone, providing further support for the idea that the screening assay has the potential to identify compounds that recognize unique conformations of the overall regulatory region. Compound **2** also bound to near-full-length Hck-YEEI, supporting the idea that this compound prefers the assembled conformation of the kinase. Compound **1** showed very similar binding characteristics as compound **2**; compounds **3** and **4** also selectively bound to the multi-domain Hck proteins but with higher K_D_ values.

All four confirmed hit compounds displaced the VSL12 peptide probe from the Hck U32L protein with 2-digit μM potency in the FP assay (Figure 3). K_D_ values observed with this protein and each compound by SPR were of the same order of magnitude (30–55 μM), with the exception of compound **4** which yielded a K_D_ value of 831 μM. The weaker affinity displayed by compound **4** in the SPR assay may reflect differences in access of this particular compound to the U32L protein in solution vs. the surface of the biosensor chip.

To investigate selectivity, we next performed SPR experiments with a similar set of proteins derived from the Src-family member, Fgr. Like Hck, Fgr is also expressed in hematopoietic cells of myeloid lineage and high-level expression has been linked to the pathogenesis of AML (Shen et al., 2018; Weir et al., 2018). Three Fgr proteins were expressed and purified for SPR, including a dual SH3-SH2 domain protein, the complete regulatory region, consisting of the unique, SH3, and SH2 domains plus the SH2-kinase linker (U32L protein) as well as near-full-length Fgr, lacking only the unique domain and encoding the same modified C-terminal tail as near-full-length Hck (Fgr-YEEI). With Fgr, a different pattern of binding was observed for the four pyrimidine diamines. Compounds **1** and **2** bound to all three Fgr proteins, although with lower affinity for Fgr U32L than for Hck, while compound **3** did not bind to any of the Fgr proteins. Compound **4**, on the other hand, showed enhanced binding with all three Fgr proteins compared to Hck. Overall, these results identify this pyrimidine diamine scaffold as a potential allosteric ligand for Hck and other Src-family kinases, and suggest that modifications to this scaffold may afford enhanced potency and specificity for individual family members.

### Docking Studies Identify Potential Binding Sites for Pyrimidine Diamine Ligands at the Hck SH3-Linker Interface

**Figure 6:**
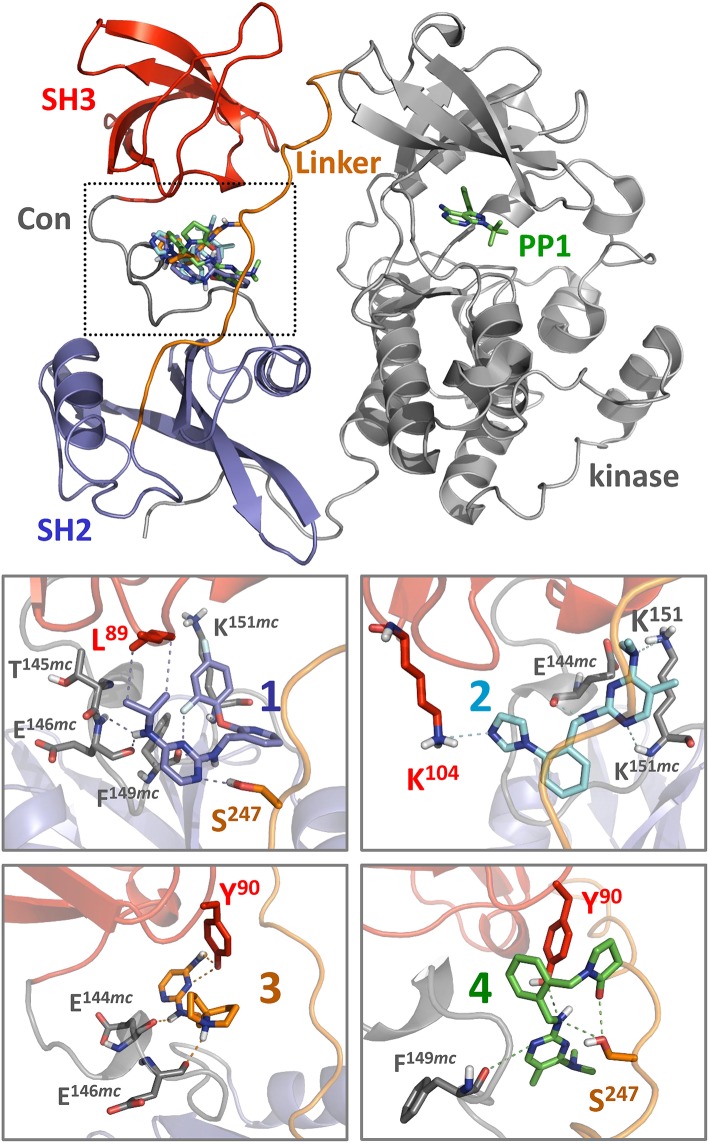
Docking predicts binding of hit compounds to the Hck regulatory region. Compounds **1** through **4** from the Hck-U32L screen (Figure 4) were docked to the crystal structure of near-full-length Hck (PDB: 1QCF) using Smina (Koes et al., 2013). The overall near-full-length Hck structure is shown at the top including the kinase domain, where the ATP-competitive inhibitor PP1 marks the active site. All four compounds are predicted to bind within a pocket formed by the convergence of the SH3 domain, the SH3-SH2 connector (*Con)*, and the SH2-kinase linker (boxed region). Close-up views of binding sites for compounds **1** through **4** (lower panels) show predicted interatomic contacts between the ligand and side/main chain residues ≤ 4 Å (*dotted lines*). Side chains and main chains (*mc*) predicted to participate in ligand binding are labeled. Nitrogen atoms present in the pyrimidine diamine core found in all four compounds make multiple contacts with the target protein. Compound **2** is unique among the four compounds, in that it makes predicted polar contacts with the side chain of SH3 Lys104 and the side and main chains of linker Lys151. This observation may relate to its greater stimulatory activity in the kinase activity assay compared to the other three compounds (Figure 7).

Each of the small molecule ligands shown in Figure 4 were docked onto the X-ray crystal structure of near-full-length Hck (PDB: 1QCF) using the application Smina (Koes et al., 2013), a refinement of AutoDock Vina previously used by our group to interrogate binding sites for competitive inhibitors and allosteric ligands for other non-receptor protein-tyrosine kinases (Grover et al., 2015; Moroco et al., 2015). Docking of all four compounds revealed a predicted binding pocket formed by the juxtaposition of the SH3 domain, the SH3-SH2 connector, and the SH2-kinase linker that is distinct from the ATP-binding site in the kinase domain (Figure 6, top). Interestingly, comparison of the lowest energy poses of each compound reveals alternative orientations and ligand-protein contacts, providing a possible explanation for differences in compound potency and selectivity (Figure 6, lower panels). Nitrogen atoms present in the pyrimidine diamine moiety shared by all four compounds accept hydrogen bonds from both side and main chain residues in this binding pocket. These docking models are consistent with the SPR results, where ligand binding was only observed with recombinant Hck proteins containing the SH3-SH2-linker region and not the individual SH3 or SH2 domains. X-ray crystallography of this region of Hck in complex with these ligands will be required to fully address this possibility.

### Pyrimidine Diamine Ligands Perturb Near-Full-Length Hck Activity *in vitro*

Biophysical and computational data presented above demonstrate that the pyrimidine diamines discovered using our FP-based HTS assay represent allosteric ligands for the regulatory SH3-SH2-linker region of Hck. These findings raise the question of the potential impact of these compounds on Hck kinase activity. To address this question, we used recombinant, near-full-length Hck-YEEI and the Z'Lyte *in vitro* kinase assay. Previous studies have shown that this form of Hck is singly phosphorylated on the negative regulatory tail tyrosine, and adopts the assembled, inactive conformation shown in Figure 6. Addition of peptide ligands that bind to the SH3 domain, the SH2 domain, or both perturb their regulatory interactions with the kinase domain, resulting in increased kinase activity (Moroco et al., 2014, 2015; Shen et al., 2018). This system provides an ideal platform to address the question of whether our pyrimidine diamine compounds have an allosteric effect on the Hck kinase domain.

**Figure 7:**
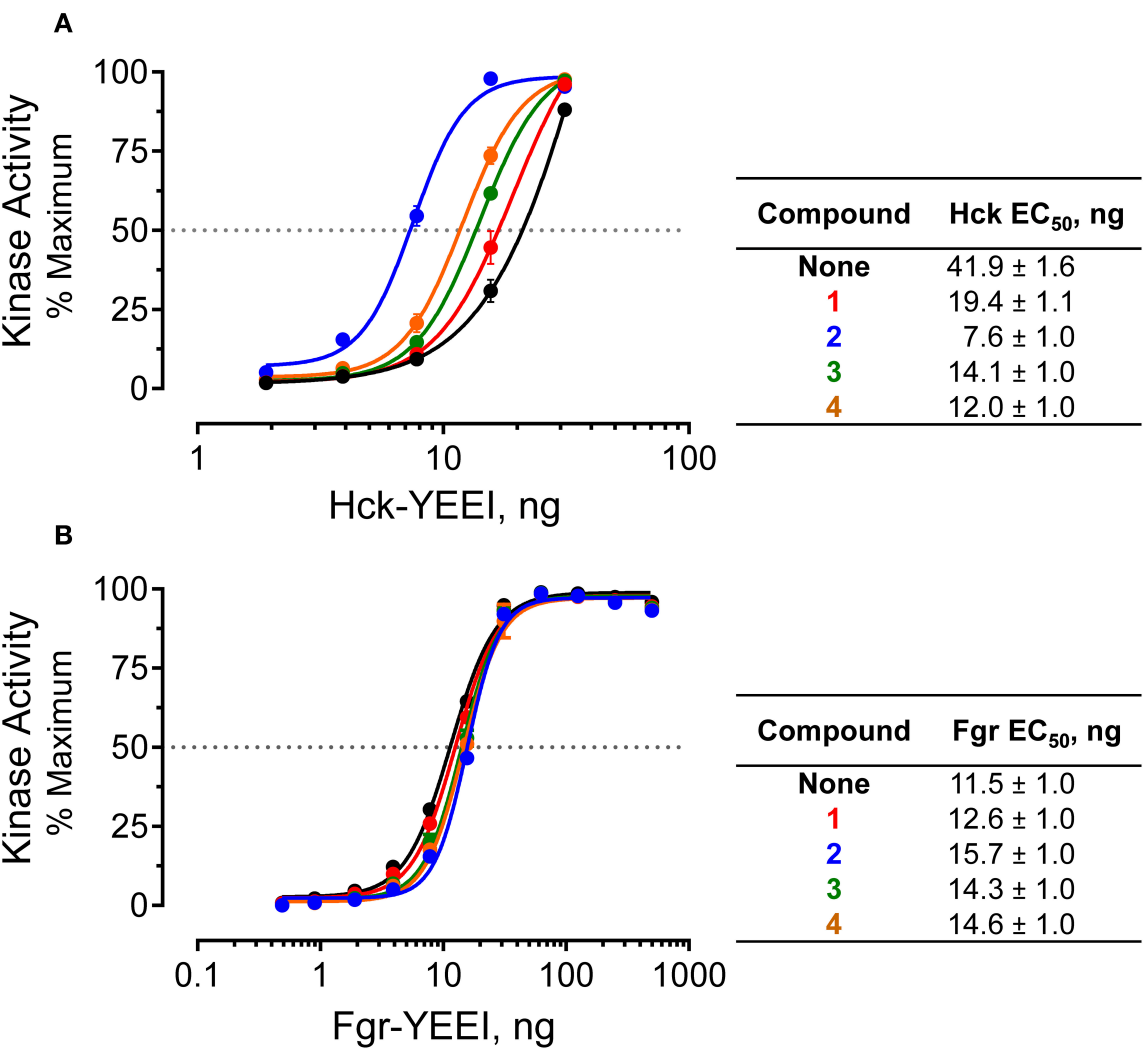
Selective activation of near-full-length Hck by allosteric modulators *in vitro*. Recombinant, near-full-length Hck **(A)** and Fgr **(B)** were assayed for kinase activity using the Z'Lyte kinase assay over the range of input kinase protein amounts shown. Assays were performed in the absence (None) or the presence of each of hit compound (**1** through **4** at a final concentration of 50 μM). Each data point was assayed in quadruplicate, and is shown as percent of maximum substrate peptide phosphorylation ± SD. The activation curves were fit by non-linear regression analysis, and the resulting EC_50_ values ± S.E. are summarized in the tables at right.

The Z'Lyte kinase assay is based on phosphorylation of a FRET-peptide substrate labeled with coumarin and fluorescein on its N- and C-termini, respectively, which form a FRET pair (Trible et al., 2006). After incubation with the kinase and ATP, the reaction is developed with a site-specific protease that selectively cleaves the unphosphorylated peptide. Cleavage results in loss of the FRET signal, and the amount of substrate phosphorylation is calculated as the ratio of coumarin to FRET fluorescence relative to a maximally phosphorylated control peptide. Kinase reactions were set up in the presence or absence of a fixed concentration of compounds **1** through **4** over a range of kinase concentrations. Following incubation, the extent of phosphorylation was plotted as a function of the amount of Hck added to each reaction, and the resulting activation curves were fitted by non-linear regression analysis. In the absence of compounds, Hck activation was best-fit by a sigmoidal activation curve, yielding an EC_50_ value of 41.9 ng/well (amount of Hck required for half-maximal activity, Figure 7A). All four pyrimidine diamines shifted the curve to the left, indicative of kinase activation. Compound **2** produced the largest increase in activity, resulting an EC_50_ value of 7.6 ng/well, while the other compounds produced intermediate effects. Using a very similar assay approach, we have shown previously that protein and peptide ligands for the Hck SH3 and SH2 domains also increase kinase activity across multiple kinase input amounts (Trible et al., 2006; Emert-Sedlak et al., 2009; Moroco et al., 2014, 2015; Shen et al., 2018). These findings suggest that the small molecules identified in the FP screen interact with near-full-length Hck in solution, leading to increased kinase activity through a related regulatory domain-displacement mechanism.

As a negative control, we performed similar experiments with an analogous form of recombinant, near-full-length Fgr. Unlike Hck, recent work has shown that Fgr is constitutively active in cells and is not susceptible to domain-displacement activation by peptide ligands for its SH3 and SH2 domains *in vitro*, despite adopting a similar assembled conformation of its SH3, SH2 and kinase domains (Shen et al., 2018). Addition of the pyrimidine diamine ligands had no significant effect on Fgr activity in the Z'Lyte kinase assay, despite the ability of three of these compounds to interact with near-full-length Fgr by SPR (Figure 7B). These results are consistent with published data using SH2 and SH3 peptide ligands, which also had no effect on Fgr kinase activity despite binding to their respective regulatory domain targets by SPR analysis (Shen et al., 2018).

## Summary

Here we report the development of an automated HTS assay for the discovery of small molecule modulators of the Src-family kinase, Hck. Using this assay, we identified a series of ligands for the Hck regulatory region that share a common pyrimidine diamine scaffold based on their ability to inhibit the FP signal resulting from the binding of a peptide probe to a Hck U32L target protein. While their potency for Hck is relatively modest, with K_D_ values in the low to mid-micromolar range, binding was dependent upon and most avid for the Hck target protein consisting of the complete regulatory region, with no binding observed to the individual SH3 and SH2 domains. This observation suggests that these ligands recognize the Hck regulatory region in a conformation-dependent manner, a conclusion supported by computational docking models using the crystal structure of near-full-length Hck in the inactive, assembled state. Structures of near-full-length Hck and Src suggest that all Src-family members adopt similar assembled conformations in the downregulated state (Schindler et al., 1999; Xu et al., 1999). However, the significant sequence differences present in their regulatory domains and the interfaces between them are likely to allow development of selective ligands for individual kinases. This conclusion is borne out by SPR evidence showing significant differences in the affinities of the pyrimidine diamines for the regulatory U32L proteins derived from Hck vs. Fgr. Such compounds may work in concert with existing ATP-site inhibitors to enhance therapeutic targeting of these kinases in the context of myeloid leukemia or other forms of cancer. Combinations of allosteric modulators and ATP-site inhibitors show promise in the suppression of acquired resistance that is often observed when ATP-site inhibitors are used as single agents (Wylie et al., 2017).

## Data Availability Statement

This manuscript contains previously unpublished data. All data are presented in the article/Supplementary Material.

## Author Contributions

TS and PJ conceived and designed the study. HD and DC performed the laboratory experiments. BW and CC performed the computational docking studies. TS wrote the manuscript with editorial input from HD, PJ, and CC.

### Conflict of Interest

The authors declare that the research was conducted in the absence of any commercial or financial relationships that could be construed as a potential conflict of interest.
