# Supplementary material for: Discovery of Non-peptide Small Molecule Allosteric Modulators of the Src-family Kinase, Hck
Source: Front Chem. 2019 Nov 28;7:822. doi: 10.3389/fchem.2019.00822 (PMC6893557; doi:10.3389/fchem.2019.00822)
Supplement: Supplementary file 1 [file Data_Sheet_1.PDF]

Supplementary Information

Discovery of Non-peptide Small Molecule Allosteric Modulators of the Src-family Kinase, Hck

Heather R. Dorman, David Close, Bentley M. Wingert, Carlos J. Camacho, Paul A. Johnston, and Thomas E. Smithgall

Contents

Figure S1. Titration of FP peptide probe in the presence and absence of the Hck-U32L target.

Figure S2. Titration of the Hck-U32L target protein concentration at a fixed FP peptide probe concentration

Figure S3. Five-plate assay performance test.

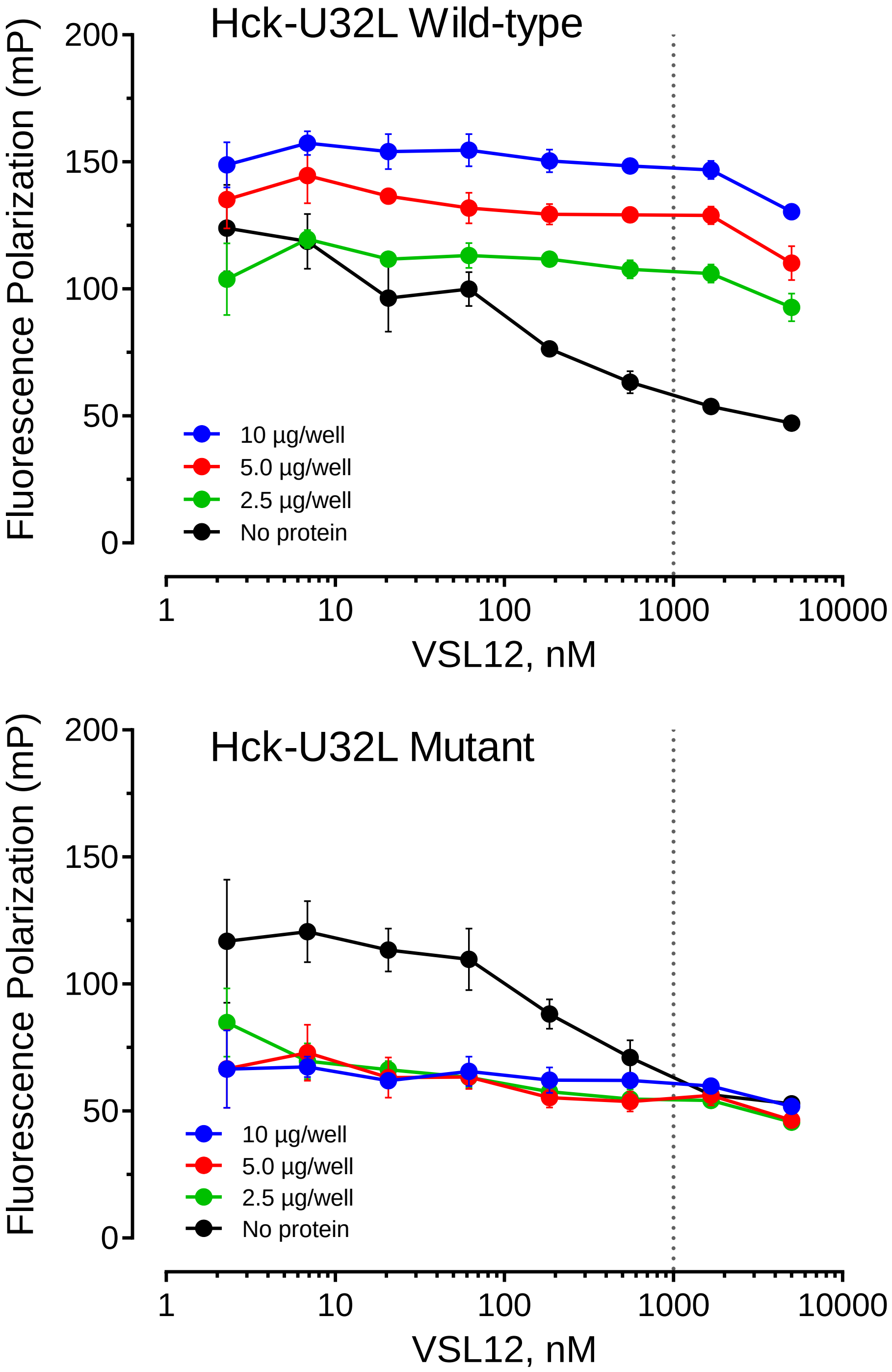

Figure S1. Titration of FP peptide probe in the presence and absence of the Hck-U32L target. FP assays were run over the range of VSL12 FP probe peptide concentrations shown in either the absence or presence of the wild-type Hck-U32L target protein (top) or the SH3 domain mutant that does not bind to VSL12. Three inputs of each Hck protein were included in this experiment as indicated by the different colors or in the absence of the target protein. Based on these results, the final peptide probe concentration used for screening was 1.0 µM (indicated by the vertical dotted line in each graph). Final assay volumes were 20 µL. Each data point represents the average FP signal ± SD for four measurements.

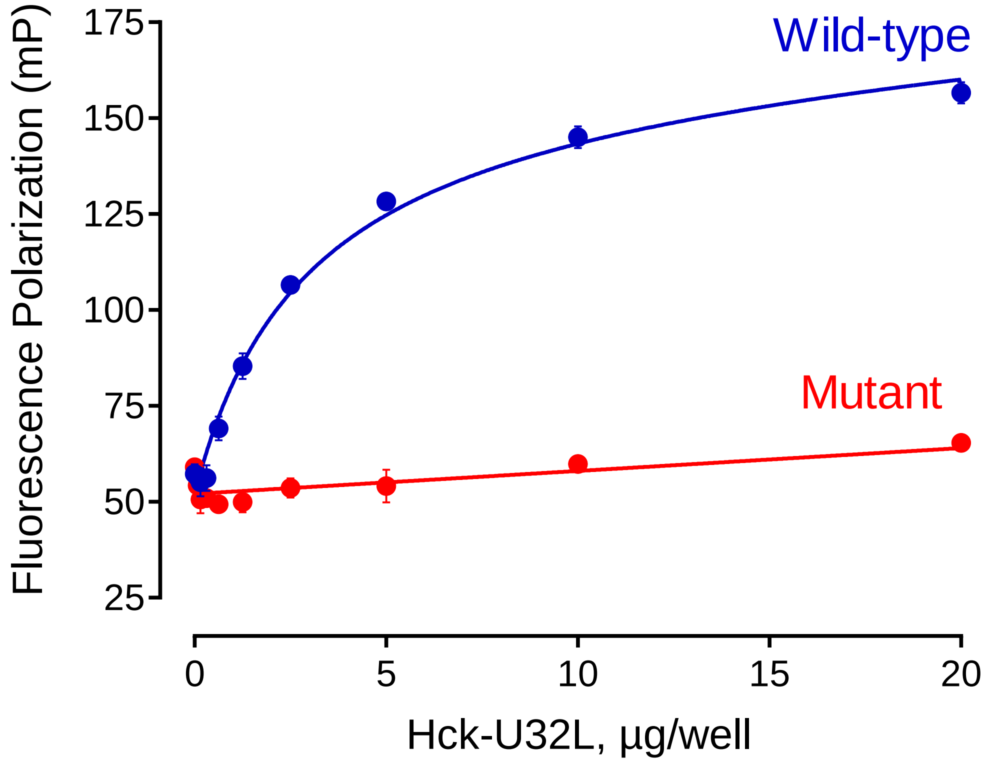

Figure S2. Titration of the Hck-U32L target protein concentration at a fixed FP peptide probe concentration. FP assays were performed with wild-type Hck-U32L target protein (blue curve) or the SH3 domain mutant that does not bind to VSL12 (red curve) over the range of protein inputs shown. The VSL12 FP peptide probe concentration was held constant at 1 µM per well. Final assay volumes were 20 µL. Based on this result, the final amount of Hck-U32L protein used for HTS was 5.0 µg per well, which represented a good compromise between signal to background ratio and overall recombinant protein requirement for the screen (approximately 500 mg of recombinant Hck-U32L protein were consumed in assay development and screening). Each data point represents the average FP signal ± SD for four measurements.

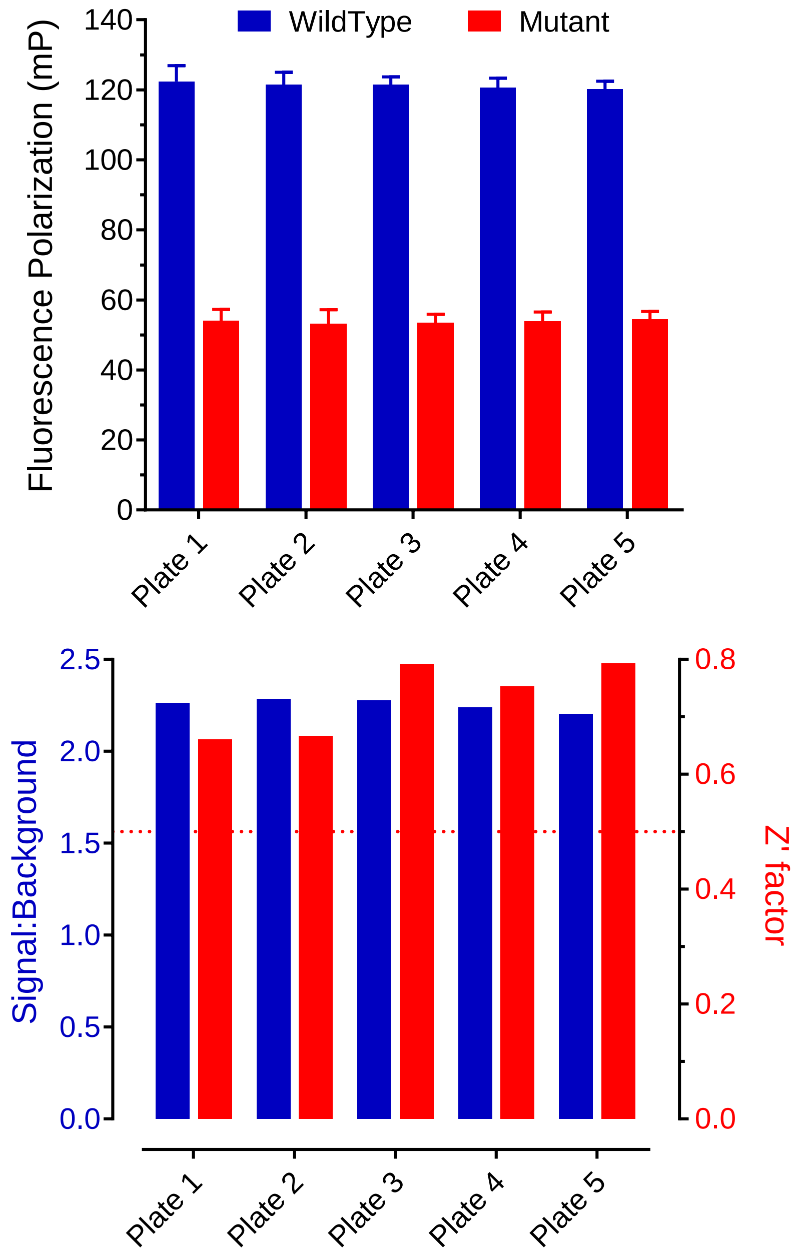

Figure S3. Five-plate assay performance test. The automated Hck-U32L FP assay was validated using five identical 384-well plates with the maximum control (n=32) and DMSO-treated (n=320) wells containing the wild-type Hck-U32L target protein, and the minimum control (n=32) wells containing the SH3 domain mutant that does not bind to the VSL12 probe peptide. All wells contained 1% DMSO, the final concentration used in the compound screen. The average FP signal ± SD (n=32) for maximum and minimum plate control sets are shown at the top, while the signal to background ratio from the wild-type vs. mutant protein wells along with the Z' factor coefficient for each plate are shown in the lower panel. Each well contained 5.0 µg of Hck-U32L protein and a final VSL12 FP peptide probe concentration of 1.0 µM; the conditions used for the 60,000 compounds HTS campaign described in the main text. All assay plates passed our quality control criteria, and the normalized percent inhibition data from the 1600 wells of the 5 x 384-well DMSO plates closely approximated a normal distribution (data not shown) with none of the wells exhibiting ≥ 50% inhibition of the Hck-U32L FP signal, producing an estimated false positive rate of 0%. An analysis of variance (ANOVA) in the DMSO validation data revealed no significant row/column effects or other positional biases (data not shown).
